# Proteomic Responses to Drought Vary Widely Among Eight Diverse Genotypes of Rice (*Oryza sativa*)

**DOI:** 10.3390/ijms21010363

**Published:** 2020-01-06

**Authors:** Sara Hamzelou, Dana Pascovici, Karthik Shantharam Kamath, Ardeshir Amirkhani, Matthew McKay, Mehdi Mirzaei, Brian J. Atwell, Paul A. Haynes

**Affiliations:** 1 Department of Molecular Sciences, Macquarie University, North Ryde, NSW 2109, Australia; sara.hamzelou@hdr.mq.edu.au (S.H.); dana.pascovici@mq.edu.au (D.P.); karthik.kamath@mq.edu.au (K.S.K.); ardeshir.amirkhani@mq.edu.au (A.A.); matthew.mckay@mq.edu.au (M.M.); mehdi.mirzaei@mq.edu.au (M.M.); 2 Australian Proteome Analysis Facility, Macquarie University, North Ryde, NSW 2109, Australia; 3 Department of Biological Sciences, Macquarie University, North Ryde, NSW 2109, Australia; brian.atwell@mq.edu.au

**Keywords:** rice, drought stress, shotgun proteomics, label-free quantitation, mass spectrometry

## Abstract

Rice is a critically important food source but yields worldwide are vulnerable to periods of drought. We exposed eight genotypes of upland and lowland rice (*Oryza sativa* L. ssp. *japonica* and *indica*) to drought stress at the late vegetative stage, and harvested leaves for label-free shotgun proteomics. Gene ontology analysis was used to identify common drought-responsive proteins in vegetative tissues, and leaf proteins that are unique to individual genotypes, suggesting diversity in the metabolic responses to drought. Eight proteins were found to be induced in response to drought stress in all eight genotypes. A total of 213 proteins were identified in a single genotype, 83 of which were increased in abundance in response to drought stress. In total, 10 of these 83 proteins were of a largely uncharacterized function, making them candidates for functional analysis and potential biomarkers for drought tolerance.

## 1. Introduction

Rice, corn, and wheat are the three primary crops cultivated for human consumption, contributing 60% of humankind’s dietary energy requirements [1]. As for all the major crops, the productivity of upland and rainfed rice is subject to frequent and unpredictable shortages of water, exacerbated by the effects of heat and salinity. Because of its evolutionary origins as a wetland species, rice is unsurprisingly considered to be one of the most susceptible crops to drought stresses and high yield losses have been reported [2]. However, there is evidence for a degree of drought tolerance in upland genotypes [3], resulting in the prospect of assuring yields in environments with an unreliable water supply. Improved tolerance to transient droughts during vegetative development is required to secure yields, particularly in the arc of upland growing regions that includes the sub-continent, China, and Southeast Asia [4].

Depending on the accession, developmental stage, and environmental factors, the response to drought stress varies [5]. Although the genome of *Oryza sativa* has been sequenced [6], a vast number of proteins have unknown metabolic functions [7]. Studying the expressed proteome of different genotypes of rice at a specific growth stage under environmental stress enables us to achieve two goals. First, we can better understand the molecular response of rice to water deficits, and additionally, we can identify variations in the response of individual accessions to drought.

Droughts and increasingly marginal environments will require molecular breeding in order to sustain the essential increase in the productivity of rice. The high number of genes involved in drought resistance and variable patterns of drought duration and severity amplify the challenge. Thus, a successful molecular breeding program requires a deeper knowledge of the differentially expressed genes that will come from interrogating diverse germplasm using transcriptomics and proteomics [8].

We examined eight genotypes of rice from different genetic backgrounds and geographic locations to quantify the interaction between extended drought and genetics on the proteome of leaves. This included combinations of both sub-species (*japonica* and *indica*), upland and lowland ecotypes, commercial cultivars, and landraces. Reputations for heat and drought stress tolerance in rice often come from reproductive physiology (e.g., pollen viability) while this study focuses on vegetative tissues. A proteomics approach builds upon previous transcriptomic studies [9,10,11,12], ultimately as a means of finding candidate marker genes for drought tolerance. The aim was to identify those proteins that respond to drought stress in all genotypes, as well as proteins that are only differentially expressed in drought-tolerant or -sensitive genotypes.

## 2. Results

### 2.1. Label-Free Shotgun Proteomic Data Analysis

Eight different varieties of rice were grown to the late vegetative stage and subjected to drought stress. These included four lowland varieties, which are not known to be stress tolerant (Doongara, Mahsuri, Nipponbare, and Reiziq) and four upland varieties: IAC1131, which is drought tolerant; IDSA77 and IR2006-P12, which are tolerant to high temperatures; and N22, which is tolerant to high temperatures and drought. Plants were grown to the late vegetative stage (70 days), then the soil water content was reduced to 50% field capacity for 10 days, then watering was withheld to induce severe drought stress. Plants were harvested when morphological examination indicated that a majority of the leaves were tightly rolled, which occurred after 3 to 7 days of severe drought stress depending on the genotype.

Label-free quantitative shotgun proteomic analysis of the eight rice genotypes upon exposure to drought stress enabled high-confidence identification of 1253 non-redundant proteins across all genotypes of *O. sativa* under well-watered and droughted conditions (Supplementary Table S1). The false discovery rate (FDR) as assessed by reversed database searching was less than 1.4% at the protein identification level, and less than 0.4% at the peptide-to-spectrum matching level, in all genotypes (Table 1).

**Figure 1:**
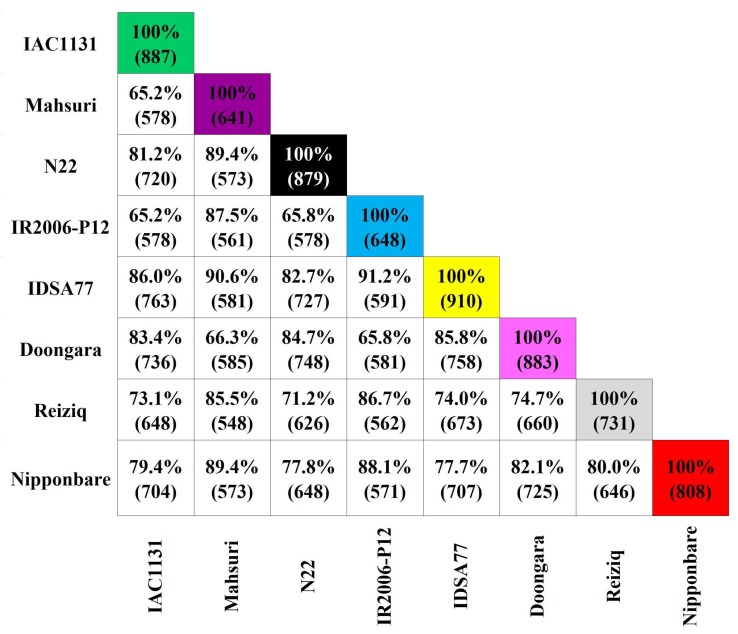
Number of proteins identified in genotypes of rice under control and stress conditions. The white squares show the pairwise overlap of the proteins identified in each genotype of rice. The diagonals show the total number of proteins identified in each genotype.

We observed that 37.9% of the identified proteins were shared across all eight genotypes (Figure 1). The largest overlap of proteins identified was observed between IDSA77 and IR2006-P12, two heat-tolerant upland *japonica* accessions, with 591 of the 648 proteins (91.2%) identified in IR2006-P12 also found in IDSA77. In contrast, the smallest overlap of common proteins was observed between IAC1131 (a Brazilian *japonica* landrace) and each of IR2006-P12 and Mahsuri (Asiatic genotypes that include *indica* genetics). Of the 887 proteins identified in IAC1131, 578 (65.2%) were identified in Mahsuri, and a slightly different set of 578 proteins (65.2%) were identified in IR2006-P12.

### 2.2. Proteins Induced in Response to Drought Stress in All Eight Genotypes

**Figure 2:**
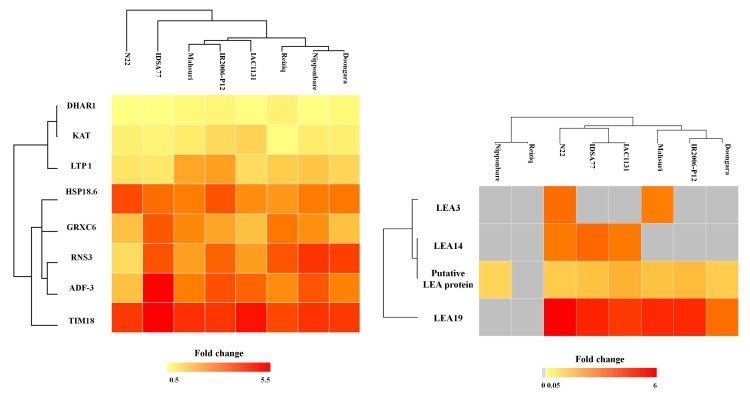
Globally upregulated proteins’ and late embryogenesis abundant (LEA) proteins’ expression pattern in drought stress. (**a**) Eight stress-responsive proteins significantly increased in abundance in all genotypes under drought stress. (**b**) Four members of LEA proteins significantly altered in response to drought stress. The fold change was calculated from the ratio of the average NSAF value in drought stress to the average NSAF value in the control condition for each of the proteins in each of the eight genotypes indicated. No protein was identified in the gray boxes. Heat maps were generated in Perseus (v. 1.6.0.2) using the log 2 NSAF fold changes [13]. Hierarchical clustering was performed using Euclidean as the distance metric and average as the linkage criterion.

Eight stress-responsive proteins significantly increased in abundance in all genotypes under drought stress: DHAR1, KAT, LTP1, HSP 18.6, GRXC6, RNS3, ADF3, and TIM18 (Figure 2). DHAR1 is GSH-dependent dehydroascorbate reductase 1, KAT is a 3-ketoacyl-CoA thiolase-like protein (KAT-like), LTP1 is a non-specific lipid-transfer protein 1, HSP 18.6 is a small heat shock protein, GRCX6 is Glutaredoxin C6, ADF3 is actin-depolymerizing factor 3, RNS3 is ribonuclease 3, and TIM18 is an 18 kDa putative mitochondrial import inner membrane translocase subunit.

Q10MK4 is an 18 kDa putative mitochondrial import inner membrane translocase subunit (TIM) family member, and in this study, it was found to be one of the most significantly changed proteins across all genotypes under drought stress, with an average fold change of 24.6. The greatest fold change in response to drought stress for both TIM18 and GRXC6 was detected in IDSA77 while these proteins were least affected by the drought treatment in N22 (Figure 2). Ribonuclease 3 (RNS3) showed the greatest increase in abundance in response to drought stress in Nipponbare and Doongara but was also increased significantly in the other genotypes examined.

Even though 18.6 kDa class III heat shock protein (HSP18.6) was upregulated significantly across all genotypes in the stress conditions, the largest changes in abundance were reported in the three most heat-tolerant genotypes, namely IDSA77, N22, and IR2006-P12. Non-specific lipid-transfer protein 1 (LTP 1), 3-ketoacyl-CoA thiolase-like protein (KAT-like), and GSH-dependent dehydroascorbate reductase 1 (DHAR1) also increased in abundance but with a smaller fold change in the stress condition across all genotypes.

### 2.3. Proteins Induced in Response to Drought Stress Uniquely in a Single Rice Genotype

**Figure 3:**
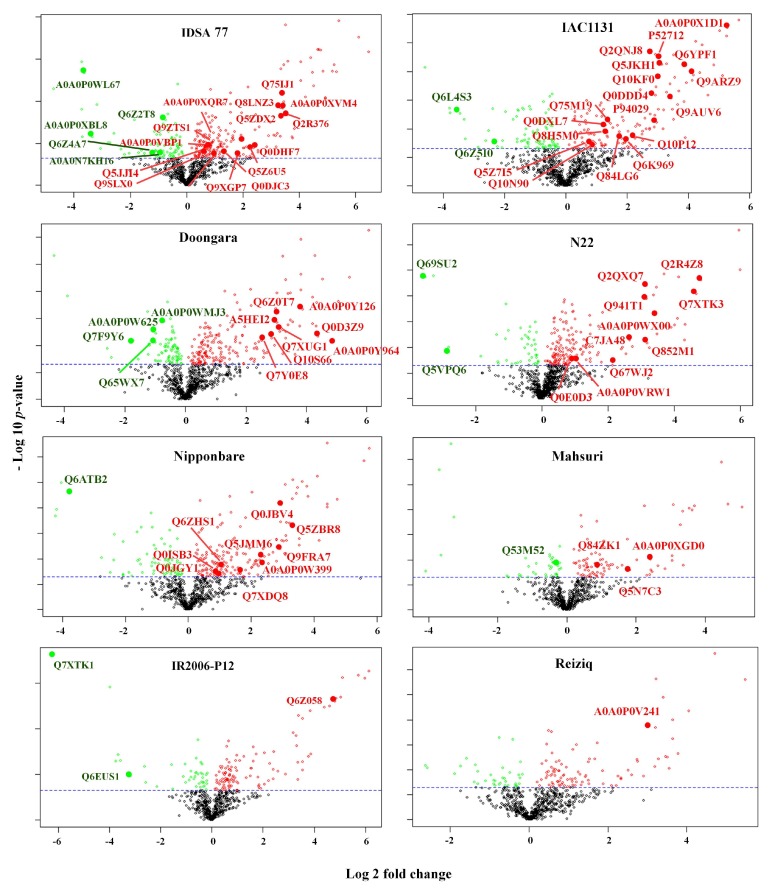
Volcano plots for all unique stress-responsive proteins identified in rice genotypes. Each point represents a protein with an average log 2-fold change along the x-axis (log 2 of the ratio of the average NSAF value in drought stress to the average NSAF value in the control condition) and –log10 *p*-value along the y-axis. Red, green, and black points show the upregulated, downregulated, and unchanged proteins, respectively. The identifier for unique proteins is shown in each plot. Dashed line shows the *p*-value of 0.05 cut-off.

A total of 213 proteins (17% of the total identified proteins) unique to a single genotype were identified (‘unique proteins’); among these, 83 were significantly changed in abundance in response to drought stress (Supplementary Table S2). ‘Unique proteins’ induced by drought stress for each genotype are shown in the volcano plots of protein abundance and fold change for each rice genotype (Figure 3): The impact of drought was not uniform across all genotypes. The most significantly changed unique drought-response proteins were in IAC1131 and 18 in IDSA77 (20 proteins) while 12 unique stress-response proteins were identified in Doongara and N22 and 10 in Nipponbare. Most of the differentially regulated proteins in IR2006-P12, Mahsuri, and Reiziq were also identified in other genotypes, so a lower number of unique stress-responsive proteins was detected in these genotypes. In this study, a number of proteins with an uncharacterized function, or a predicted activity, increased in abundance in response to drought stress (Table 2).

### 2.4. GO Functional Classification of Significantly Altered Protein in Response to Drought

**Figure 4:**
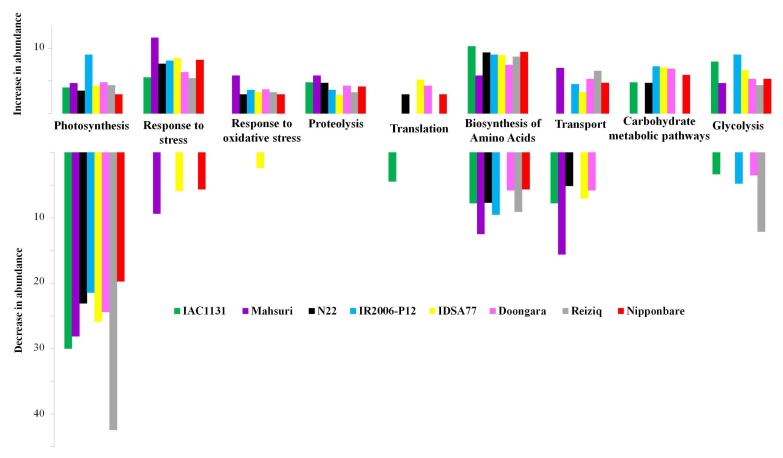
Functional classification of differentially expressed proteins in different genotypes of rice under drought conditions. The bars illustrate the percentage of proteins in nine functional categories, which are significantly increased and decreased in abundance under drought stress. Different colors represent different genotypes of rice as indicated.

Gene ontology (GO) functional classification was performed to explore the molecular function of significantly changed proteins in response to severe drought stress. The major functional categories of proteins that responded differentially in drought conditions included photosynthesis, oxidative stress response, proteolysis, and translation. The majority of proteins that decreased in abundance in all genotypes of rice in response to drought stress contribute to the photosynthesis machinery (Figure 4). More than 30% of the proteins that decreased in abundance in Reiziq and IAC1131 after a period of drought were categorized as photosynthesis-associated proteins, with many of them involved in photosystem I and II reaction centers and energy transfer. In contrast, proteins that were increased in abundance in response to drought stress were mostly involved in carbon fixation.

One of the interesting findings of this study is the increase in abundance of proteolysis-associated proteins and oxidative stress-responsive proteins in drought conditions. Among proteolysis-associated proteins, aspartic proteinase (P42211) increased in abundance in all genotypes during drought, except in IDSA77. Superoxide dismutase and ascorbate peroxidase are key enzymes involved in ROS scavenging, and these were shown to increase in abundance in drought in most of the rice genotypes. Thioredoxin H1 (OsTrxh1) is another important protein in ROS scavenging that was found in all genotypes, with a significant change in drought conditions in seven genotypes, excluding IAC1131.

### 2.5. Parallel Reaction Monitoring (PRM) Validation

**Figure 5:**
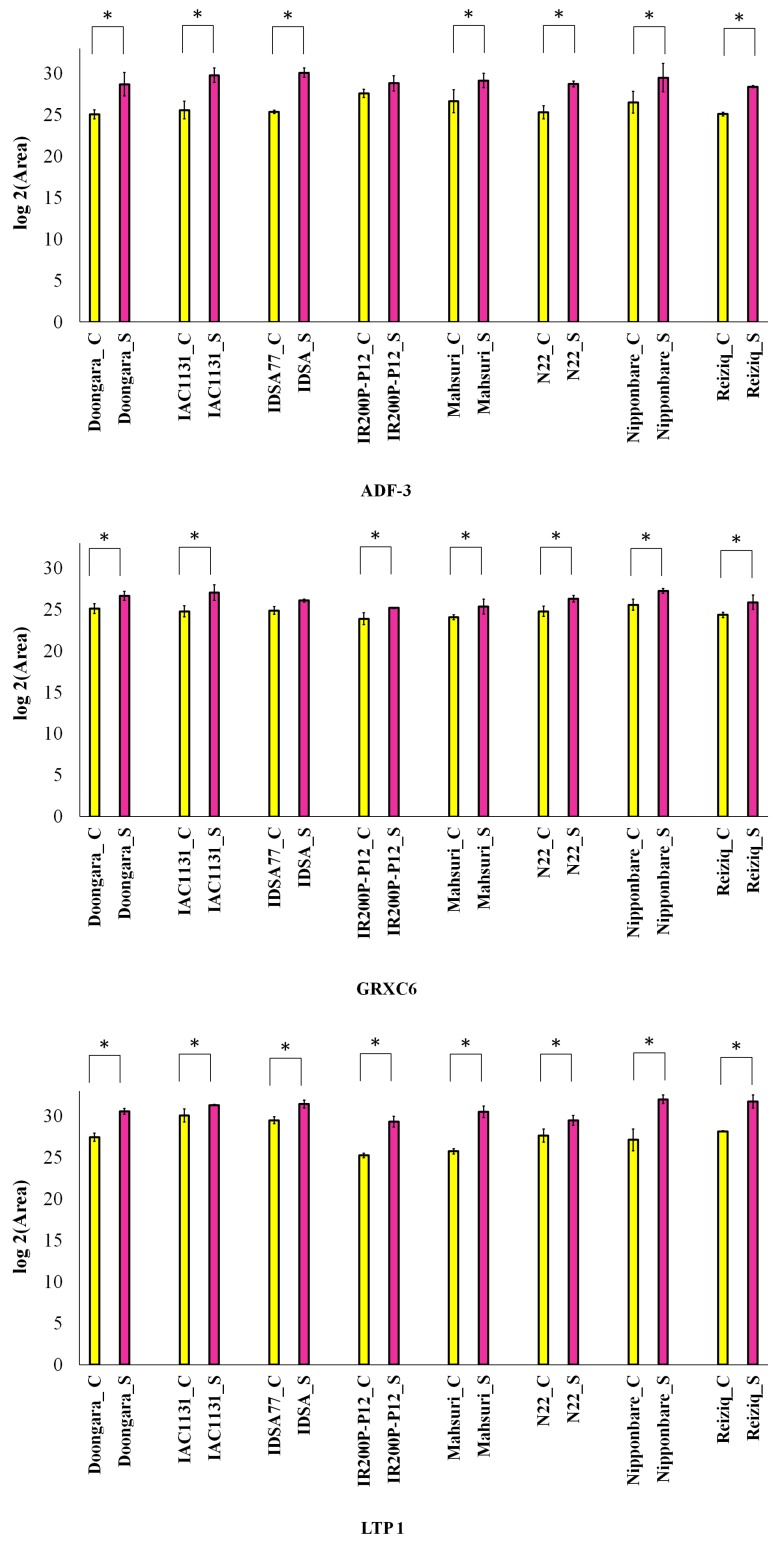
PRM verification of three proteins induced in all genotypes of rice. The bars illustrate the measured relative abundance of ADF-3, GRXC6, and LTP 1 in all eight rice genotypes under control (C) and drought stress (S) conditions, with statistically significant differences according to the Mann–Whitney U-test indicated (*p*-values < 0.05) by an asterisk (*). Error bars show the standard deviation calculated from three replicate experiments.

To verify the differential expression changes of drought stress-responsive proteins reported from label-free shotgun proteomics, parallel reaction monitoring (PRM) analysis was performed on a subset of proteins, using the same extracted peptide samples from the discovery phase study. Three differential proteins induced in response to stress in all genotypes were validated by PRM, including ADF-3, GRXC6, and LTP 1. The results obtained by PRM agree with the label-free shotgun proteomics data, showing a global increase in the abundance of the three proteins in response to drought stress (Figure 5). PRM results confirmed that the stress-induced increase in abundance of ADF-3 was greater in IDSA77 than in other genotypes of rice, which is in close agreement with the label-free quantitative shotgun proteomics data. Similarly, the PRM results also showed that for LTP1, the largest increase in abundance was in Mahsuri and Nipponbare, which is again in good agreement with the label-free quantitative shotgun proteomics data.

## 3. Discussion

We present a detailed proteomic profile of the drought-associated changes in eight genotypes of rice. These genotypes of *Oryza sativa* were chosen to reflect diverse origins (e.g., upland and lowland), with a strong emphasis on heat tolerance (e.g., N22, IDSA77, and IR2006-P12) and geographic origin (e.g., Asia, Africa, South America, and Australia). This genetic diversity was expected to be reflected in variance in the proteome when each genotype was exposed to sustained drought. Well-established physiological responses to drought include absciscic acid (ABA) signaling and stomatal aperture to reduce transpiration. In more severe drought (and other abiotic stresses), cells generate reactive oxygen species (ROS), which are naturally scavenged in protective mechanisms. Proteins that are misfolded and denatured in stressed cells are destined for proteolytic degradation. Overall, cell homeostasis under drought conditions requires broad-scale metabolic adjustments to ensure survival. This might be expected to be achieved in part through an altered abundance of key proteins.

We exposed all eight genotypes of *O. sativa* to an extended period of drought, increasing from moderate to severe, at the late vegetative stage. This experimental design enabled the identification a group of eight stress-responsive proteins found in all genotypes in response to drought stress (regardless of their level of their tolerance to drought) along with proteins that were induced in response to drought stress in most genotypes, such as late embryogenesis abundant (LEA) proteins. We also identified a number of stress-responsive proteins whose abundance changed significantly in response to drought in just one of the eight genotypes.

Naturally, the eight proteins induced in response to drought in all genotypes (DHAR1, KAT, LTP1, HSP 18.6, GRXC6, RNS3, ADF3, and TIM18) are prime candidates for the selection of drought-tolerance markers. Most of these proteins have been reported to play important roles in response to plant hormones [14,15,16,17] and are involved in detoxification of reactive oxygen species (ROS) [15,18,19]. Protein transport to mitochondria through the membrane is facilitated by TIM complexes, potentially delivering antioxidant enzymes to mitochondria and enabling ROS detoxification during water deficits. Increased abundance of this protein under drought conditions has been previously suggested to play a role in maintaining the integrity of mitochondrial membranes during stress conditions [19]. Similarly, DHAR1 plays a role in ROS detoxification, as well as protecting other proteins from aggregation due to stress conditions [18]. More generally, glutaredoxin-C6 (GRXC6) may alleviate the negative impacts of ROS while tissues are exposed to a water deficit, as it has been reported to be involved in the maintenance of the cellular redox potential [20].

Drought also induced major increases in the abundance of actin-depolymerizing factor 3 (ADF-3). The protein plays a role in de-polymerization of actin and therefore, remodeling of the cytoskeleton. Its role in the leaves of droughted plants remains unknown. While ADF-3 and other members of the ADF family, such as ADF-5 [16], have a function in abscisic acid-induced stomatal closure and therefore transpiration, it needs to be established that stomatal complexes alone could strongly influence the abundance of this group of proteins.

RNA degradation is a common response to severe drought stress in the eight rice genotypes selected. Ribonuclease 3 (RNS3) has been shown to increase in abundance in a variety of stresses, including drought, salt, and cold [21], enabling the re-programming of the transcriptome under increasingly damaging conditions.

The remaining drought-responsive proteins are also associated with metabolic stress. LTP1 responds to abiotic stress-related plant hormones, such as ABA, and plays a crucial regulatory role in plant resistance to drought stress [14]. KAT is a key enzyme in fatty acid degradation and might be involved in ABA signaling and play a role in controlling ROS production in cells [17]. Finally, small heat shock proteins are expressed in response to many environmental stresses, including drought stress [22].

Drought and heat stress often, but not always, occur simultaneously in nature and hence this paper investigated the biological response of heat-tolerant genotypes to drought stress, in order to explore the possibility of common or overlapping tolerance mechanisms. To address this, three genotypes of rice with a reputation for heat tolerance, particularly at the reproductive stage (N22, IR2006-P12, and IDSA77), were included for proteomics analysis. A number of heat shock proteins significantly increased in abundance under drought conditions in these heat-tolerant genotypes. Although small heat shock protein HSP18.6 was identified in all eight genotypes of rice, 17.9 kDa class I heat shock proteins (Hsp17.9A) was identified only in two heat-tolerant genotypes (N22 and IDSA77) while heat shock 70 kDa protein BIP5 (luminal-binding protein 5 (Q6Z058)) was only identified in IR2006-P122 (Figure 3).

The LEA protein family has been reported to be enhanced in drought tolerance by stabilizing cell membranes and protecting proteins. LEA proteins also function as chaperone proteins, protecting against cellular damage [23]. In addition to those proteins induced in response to stress in all eight genotypes, four members of the LEA gene family increased in abundance in drought stress in most genotypes examined in this study, including LEA3 (Q8S7U3), LEA14 (Q94JF2), putative LEA protein (Q75LD9), and LEA19 (P0C5A4) (Figure 2b). LEA19 was identified as the most strongly upregulated protein in response to drought in both N22 and Mahsuri, although this protein was also relatively abundant in other genotypes, including IDSA77 and IR2006-P12. The large increase in abundance of LEA3 in drought was notable in N22 and Mahsuri (11 and 8.5 NSAF fold change, respectively). Putative LEA protein was also identified as an upregulated protein in stress conditions in most of the genotypes, albeit with lower peptide fold changes (2–3.5 NSAF). Interestingly, all identified members of the LEA protein family were upregulated in the drought- and heat-adapted N22 while none were affected by drought in Reiziq, which is not known to be tolerant to stress (Figure 2b). Overexpression of LEA14 and LEA19 has been previously reported to enhance drought tolerance in rice and *Phaseolus vulgaris*, respectively [24,25]. The LEA protein family members identified in this study may have potential as biomarkers of drought stress.

Gene ontology classification of the proteins altered in the abundance in genotypes of rice in response to drought stress revealed the functional categories that were the most strongly affected. These are discussed in more detail below, and include photosynthesis, carbon metabolism, oxidative stress, glycolysis and proteolysis, and translation.

We observed a global decrease in the abundance of photosynthesis-associated proteins in all genotypes of rice, which emerge as a common response to stress conditions. GO data also showed that stress-responsive proteins, especially those involved in oxidative stress, increased in response to drought. Water deficit is known to trigger a cascade of cellular processes, which result in metabolic reprogramming in favor of energy conservation [26]. For example, photosystem II 22 kDa protein Os04g0690800 (Q0J8R9) increased strongly in abundance during drought, probably because of its important role in non-photochemical quenching and thus protection against excess irradiation. Moreover, increased expression of this protein helps chloroplastic quinone A to be more readily oxidized, which is required to act as a signal for stomatal closure [27].

Cells are exposed to dangerously high concentrations of ROS when plants undergo drought stress [28]. The undesirable impacts of ROS concentration in cells lead to degradation of biomolecules, including proteins [29]. Therefore, cells respond by increasing expression of proteins that are required to scavenge ROS and proteolytically degrade damaged proteins. Some ROS scavengers and proteolysis-associated proteins increased in abundance in almost all genotypes (e.g., thioredoxin H1 (OsTrxh1) and aspartic proteinase (P42211)). However, a number of proteins, including serine carboxypeptidase-like protein (P52712) and proteasome subunit alpha type-2 (Q10KF0), from the proteolysis-associated group and metallothionein-like protein 2A (P94029) from the ROS scavenger group were identified as genotype-specific proteins under drought stress in IAC1131. All three proteins have previously been reported to be involved in plants’ stress response [30,31,32].

Proteins involved in translation also changed in abundance in some genotypes of rice in response to drought. A significant number of differentially changed proteins identified only in IAC1131 were involved in translation and post-translational modification, including ubiquitin-40S ribosomal protein S27a-1 (Q9ARZ9), eukaryotic translation initiation factor 3 subunit Eif3f (Q75M19), eIF3c (Q84LG6), and SKP1-like protein 1 (Q2QNJ8). This may be linked to enhanced translation of stress-responsive proteins, which might be required for adaptation of cells under drought stress [33]. Most of the proteins involved in protein biosynthesis (translation), including elongation factors, tend to be upregulated under drought stress, which may be linked to the increased biosynthesis of specific stress-response proteins [34]. Our study showed that 14 different types of elongation factor proteins increased in response to stress, among them putative elongation factor 2 (Q6H4L2), identified as being the highest level of fold change in drought in IR2006-P12.

## 4. Materials and Methods

### 4.1. Plant Material and Sample Preparation

Seeds of eight genotypes of rice (described in Table 3) were grown in pots filled with 5 kg of Robertson soil. Plants were fertilized at 30 days and 60 days by addition of 250 mL of 0.5 g/L NPK—23:3.95:14 liquid fertilizer. The experiment was carried out in controlled glasshouses under a 12-h photoperiod with a light intensity of 700 μmol m^−2^ s^−1^ at 28/22 °C (day/night). Rice plants were watered to 100% field capacity (FC) for 70 days until the plants reached the late vegetative stage.

**Figure 6:**
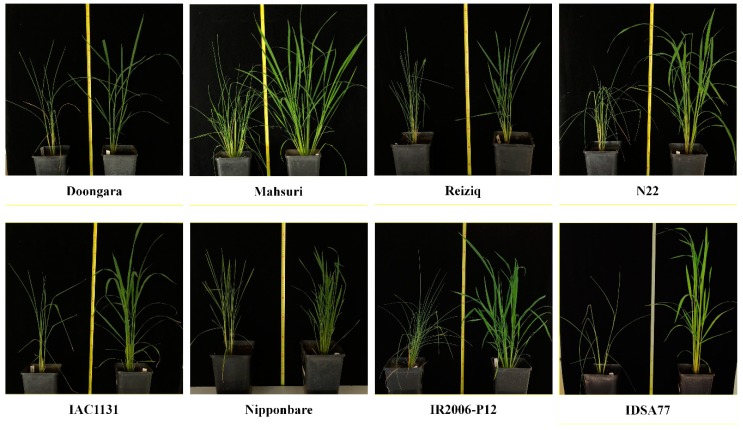
Rice genotypes used in the study under control (right) and drought stress (left) conditions. The leaves in stress conditions were rolled due to the severe drought stress.

Drought stress was imposed by withholding water until 50% FC was reached. The rate of evapotranspiration in drought-stressed plants was monitored daily by weighing the pots, followed by maintaining the soil water content at the desired level by adding the evaporated water. The stressed plants were maintained at 50% FC for 10 days. The rice plants were then exposed to a more severe stress by withholding water for at least 3 days until leaf rolling occurred, as a morphological and physiological symptom of drought stress. Due to the different usage of soil water and transpiration level in each genotype, rolled leaves were collected at different time points from 3 days to 7 days (as shown in Figure 6). Fully expanded and rolled leaves were harvested from plants under control and stress conditions, respectively. Leaf samples were immediately frozen in liquid nitrogen, and then stored at −80 °C for further analysis. All subsequent experiments were performed as biological replicates on leaf material collected from three separate plants.

### 4.2. Protein Extraction and Protein Assay

Leaf samples were ground to a fine powder in liquid nitrogen using a mortar and pestle, and the protein was extracted from 50 mg of leaf powder using the trichloroacetic acid-acetone method. Briefly, leaf powder was suspended in 1.5 mL of 10% trichloroacetic acid in acetone, 2% β-mercaptoethanol, vortexed for 30 min at 4 °C, and incubated at −20 °C for 45 min. After centrifugation of the extract, the pellet was collected and washed three times with 100% ice-cold acetone, followed by centrifugation after each washing step at 16,000× g for 30 min. The protein pellet was lyophilized in a vacuum centrifuge and resuspended in 3% SDS in 50 mM Tris-HCl (pH 8.8). Samples were then methanol-chloroform precipitated. The protein pellet was suspended in 8 M urea in 100 mM Tris-HCl (pH 8.8). The concentration of protein in the solution was measured by bicinchoninic acid (BCA) assay kit (Thermo Scientific, San Jose, CA, USA).

### 4.3. In-Solution Digestion and Peptide Extraction

Protein samples were diluted five times with 100 mM Tris-HCl (pH 8.8) to reduce the concentration of urea to 1.6 M. The samples were reduced with 10 mM dithiothreitol at 37 °C for 1 h, followed by alkylation with 20 mM iodoacetamide in the dark for 45 min at room temperature. Finally, samples were digested at 37 °C overnight with trypsin at an enzyme:protein ratio of 1:100. The reaction was stopped by the addition of formic acid to final concentration of 1%, and desalted using a SDB-RPS (3 M-Empore) Stage-tip. Samples eluted from Stage-tip using 200 μL of 80% acetonitrile/5% ammonium hydroxide. Desalted peptides were dried in a vacuum centrifuge and resolubilized in 0.1% formic acid. The concentration of peptide samples was determined by a micro-BCA assay (Pierce Biotechnology, Rockford, IL, USA).

### 4.4. Nanoflow Liquid Chromatography–Tandem Mass Spectrometry

Tryptic peptides were analyzed by nanoflow liquid chromatography–tandem mass spectrometry (nLC–MS/MS) using a Q Exactive Orbitrap mass spectrometer coupled to an EASY-nLC1000 nanoflow HPLC system (Thermo Scientific, San Jose, CA, USA). Reversed phase columns (75 µm internal diameter) were packed in-house to 10 cm with Halo C18 packing material (2.7 µm beads, 160 Å pore size, Advanced Materials Technology). Peptides were eluted from the column using a 2 h linear solvent gradient, starting with 100% Buffer A [0.1% formic acid], with steps from 0 to 40% of buffer B [99.9% (*v/v*) ACN, 0.1% (*v/v*) formic acid] over 110 min and 40% to 85% of buffer B over 10 min. Tandem mass spectrometry was performed in the data-dependent acquisition (DDA) mode with MS/MS of the top 10 most abundance precursor ions at HCD normalized collision energy of 35%. Xcalibur software (version 2.06) (Thermo, Fremont, CA) was used to perform spectral acquisition over the mass range of 400 to 1500m/z, automated peak recognition, detection of ions in the Orbitrap at a resolution of 70,000, HCD fragmentation of target ions, and dynamic exclusion of fragmented ions for 90 s.

### 4.5. Parallel Reaction Monitoring (PRM) Analysis

Parallel reaction monitoring (PRM) analysis was performed using the same tandem mass spectrometry system, for the analysis of three differentially abundant proteins induced in response to stress in all genotypes of rice. Skyline software version 4.2.0 was used for PRM analysis [35]. An inclusion list consisting of the mass to charge ratio of unique precursor peptides of interest according to the results of our quantitative proteomic analysis was created and used for PRM analysis. The inclusion list started targeted scans at a resolving power of 17,500, an AGC target of 2 × 10^5^, a maximum injection time of 100 ms, and a normalized collision energy of 30% in HCD. All peaks were manually inspected to ensure that correct ions were selected. The proteins were quantified using the summation of the peak areas for the transitions in the respective peptides and proteins. The summed fragment ion peak areas were normalized with one of the most abundant peptides belonging to ribulose bisphosphate carboxylase large chain (RuBisCO). Peak areas are reported as the average of three replicate experiments, with error bars showing the standard deviation. Prior to statistical analysis, the normalized peak areas were log2 transformed. Differentially expressed proteins were identified using the Mann–Whitney U-test to compare stress versus control condition in different genotypes.

### 4.6. Protein Identification

Raw MS data files were converted to mzXML format and processed through the global proteome machine (GPM) software version 2.2.1 of the X!Tandem algorithm, which is available from http://www.thegpm.org*. Oryza sativa* protein sequence databases containing 48,932 sequences were downloaded from Uniprot as of March 2018. MS/MS spectra for 8 genotypes belonging to *Oryza sativa* were searched against the *Oryza sativa* protein database. Common human and trypsin peptide contaminants were also included, and a reversed sequence database was analyzed to evaluate the false discovery rate. Peptide-to-spectrum matching search parameters included a peptide mass tolerance of 4 Da, a fragment mass tolerance of 20 ppm tolerance of up to one missed tryptic cleavages and K/R-P cleavages. Fixed modifications were set for carbamidomethylation of cysteine and variable modifications were set for oxidation of methionine.

### 4.7. Data Processing and Quantitation

The identified proteins from three biological replicates of each genotype for both conditions were combined to create a single protein list for each genotype. The final list of identified proteins from each genotype contained the proteins that were identified reproducibly in all three replicates of at least one condition, with a minimum peptide spectral count of 11 across all replicates in both conditions. Using the abovementioned criteria produced a high stringency dataset with a protein- and peptide-level false discovery rate (FDR) less than 1.4% and 0.4%, respectively. Protein abundance data was calculated using normalized spectral abundance factors (NSAFs) and log transformed for the following statistical analyses using the Scrappy software package [36,37]. Pairwise comparisons of the stress versus control condition in different genotypes were performed using student t-tests of the log-transformed NSAF data. Proteins with a *p*-value less than 0.05 were considered as differentially expressed [37]. Fold changes were calculated as a ratio of the logNSAF value of proteins present under drought stress to normal condition. The mass spectrometric proteomic data were deposited into the ProteomeXchange Consortium via the PRIDE [38] partner repository with the Project accession PXD016150, Username reviewer28485@ebi.ac.uk; and Password W8Kkg4w4.

### 4.8. Functional Protein Annotation

Classification of differentially expressed proteins was performed based on biological function annotations using String database version 10.5 available from https://string-db.org/. The name of differentially expressed proteins was given as a query to the String database and the corresponding protein–protein interaction information were retrieved to determine the associate functions and pathways with the protein list.

## 5. Conclusions

The paper identified key proteins and processes in the leaves of rice plants that were selected from diverse germplasm. Label-free shotgun proteomics was applied to eight accessions and protein profiles were compared in the leaves after severe drought treatment and adequately watered plants. More than 40 proteins were significantly more abundant in rice leaves subject to drought, each protein being unique to one of three heat-tolerant genotypes (IAC1131, N22, and IDSA77); these represent useful targets for novel tolerance mechanisms. IR2006-P12 (heat tolerant at the reproductive stage) only had two unique drought-responsive proteins, presumably reflecting its common phylogenetic connection to IDSA77 while in Reiziq and Mahsuri, only four proteins were uniquely increased in drought. A set of eight proteins was significantly more abundant in all eight genotypes, suggesting they might have a central role in drought tolerance. Typically, some stress-responsive proteins, such as heat shock and late embryogenesis-associated proteins, sharply increased in drought. A thorough investigation of the roles of proteins that did respond to drought in sensitive genotypes, such as Nipponbare [39], is required to determine whether they are a response to drought-induced damage, or make a contribution to drought resilience.

## Figures and Tables

**Table 1:** Summary of the peptide and protein identification data of leaf samples for eight genotypes of rice.

Row	Rice Genotype	Total Proteins	Upregulated Proteins	Downregulated Proteins	Protein FDR (%)	Peptide FDR (%)
1	Doongara	883	192	87	1.12	0.02
2	IAC1131	887	129	91	1.11	0.32
3	IDSA77	910	214	85	0.07	0.02
4	IR2006-P12	648	112	43	1.36	0.05
5	Mahsuri	641	86	39	1.53	0.06
6	N22	879	171	42	1.12	0.03
7	Nipponbare	808	105	70	0.98	0.37
8	Reiziq	731	93	35	1.08	0.23

**Table 2:** Proteins with uncharacterized or predicted function, which increased in abundance in response to drought stress in only one rice genotype.

Row	Rice Genotype	Uniprot ID	Protein Name	NSAF Fold Change	Homologous Protein	Identity (Percent)
1	IDSA77	Q2R376	Expressed protein (Os11g0533400 protein)	12.03	-	-
2	IDSA77	Q0DHF7	Os05g0468800 protein	5.19	putative cold regulated protein [*Oryza sativa japonica* Group]	89%
3	IDSA77	A0A0P0XQR7	Os09g0535900 protein	2.34	DNA-(apurinic or apyrimidinic site) lyase 2 isoform X2 [*Panicum miliaceum*]	88%
4	IDSA77	Q0DJC3	Os05g0301700 protein	2.3	cytochrome c1-2, heme protein, mitochondrial [*Oryza sativa japonica* Group]	100%
5	IDSA77	A0A0P0VBP1	Os01g0895600 protein	1.8	calreticulin-3 [*Oryza sativa Japonica* Group]	100%
6	IAC1131	Q0DDD4	Os06g0232100 protein	7.62	probable serine/threonine-protein kinase SIS8	100%
7	IAC1131	Q8H5M0	Os07g0585000 protein	2.55	calcium-dependent lipid-binding (CaLB domain) family protein [*Zea mays*]	75%
8	Mahsuri	A0A0P0XGD0	Os08g0425800 protein	5.39	-	-
9	Reiziq	A0A0P0V241	Os01g0332900 protein	8.19	Predicted acidic leucine-rich nuclear phosphoprotein 32-related protein [*Oryza brachyantha*]	84%
10	N22	Q2QXQ7	Expressed protein (Os12g0147200 protein)	2.09	-	-

**Table 3:** Details of rice genotypes analyzed in this study.

Row	Rice Genotype	Ecosystem	Description
1	Doongara	Lowland	Australian japonica
2	IAC1131	Upland	*japonica*, drought tolerant
3	IDSA77	Upland	Tolerant to high temperature
4	IR2006-P12	Upland	*indica*, tolerant to high temperature
5	Mahsuri	Lowland	Indian traditional rice genotype, drought sensitive
6	N22	Upland	*indica*, tolerant to high temperature and drought
7	Nipponbare	Lowland	*japonica*, drought sensitive
8	Reiziq	Lowland	Australian *japonica*

## Supplementary Materials

Supplementary materials can be found at https://www.mdpi.com/1422-0067/21/1/363/s1.

## Abbreviations

ABA	Abscisic acid
FDR	False discovery rate
GO	Gene ontology
LEA	Late embryogenesis abundant
nLC–MS/M	nanoflow liquid chromatography–tandem mass spectrometry
NSAF	Normalized spectral abundance factor
PRM	Parallel reaction monitoring
ROS	Reactive oxygen species
